# Covariation of brain and skull shapes as a model to understand the role of crosstalk in development and evolution

**DOI:** 10.1111/ede.12421

**Published:** 2022-11-14

**Authors:** Andrew J. Conith, Sylvie A. Hope, R. Craig Albertson

**Affiliations:** ^1^ Biology Department University of Massachusetts Amherst Amherst Massachusetts USA

**Keywords:** bone, brain, development, geometric morphometrics, phenotypic integration

## Abstract

Covariation among discrete phenotypes can arise due to selection for shared functions, and/or shared genetic and developmental underpinnings. The consequences of such phenotypic integration are far‐reaching and can act to either facilitate or limit morphological variation. The vertebrate brain is known to act as an “organizer” of craniofacial development, secreting morphogens that can affect the shape of the growing neurocranium, consistent with roles for pleiotropy in brain–neurocranium covariation. Here, we test this hypothesis in cichlid fishes by first examining the degree of shape integration between the brain and the neurocranium using three‐dimensional geometric morphometrics in an F_5_ hybrid population, and then genetically mapping trait covariation using quantitative trait loci (QTL) analysis. We observe shape associations between the brain and the neurocranium, a pattern that holds even when we assess associations between the brain and constituent parts of the neurocranium: the rostrum and braincase. We also recover robust genetic signals for both hard‐ and soft‐tissue traits and identify a genomic region where QTL for the brain and braincase overlap, implicating a role for pleiotropy in patterning trait covariation. Fine mapping of the overlapping genomic region identifies a candidate gene, *notch1a*, which is known to be involved in patterning skeletal and neural tissues during development. Taken together, these data offer a genetic hypothesis for brain–neurocranium covariation, as well as a potential mechanism by which behavioral shifts may simultaneously drive rapid change in neuroanatomy and craniofacial morphology.

## Research highlights

Covariation among traits can arise due to shared functional, developmental, and/or genetic underpinnings. We measure and detect covariation between the brain and the neurocranium in an F_5_ hybrid population of cichlid fish. By mapping these traits, we uncover a pleiotropic locus that may underlie covariation and provide a means to modulate behavior and head shape simultaneously.

## Graphical abstract

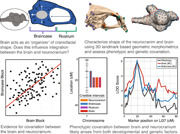

## 1 INTRODUCTION

Development is a hierarchical process that depends on the dynamic feedback among and across cells, tissues, and structures to shape the phenotype. Feedback among such structures produces measurable patterns of covariation, and the strength of this covariation is determined by spatially and temporally overlapping developmental factors (Hallgrímsson et al., 2009; Klingenberg, 2010). For example, structures in close spatial proximity may be subject to comparable developmental environments, as morphogens diffuse into adjacent cells and tissues, strengthening the association among cells within the surrounding field (Choe & Crump, 2014; Green et al., 2017; Hu et al., 2015). Similarly, temporally modulating cellular migration (e.g., neural crest migration) can have large consequences for how seemingly distinct structures may covary (Sutton et al., 2021). Characterizing covariation among distinct structures in an organism provides a window into how spatially and temporally overlapping developmental processes interact through ontogeny. By examining where and when covariation arises and persists both within and across levels of biological organization (i.e., cells, tissues, organs, structures), we can appreciate the genetic, developmental, and evolutionary implications of trait integration.

Covariation among structures can arise even between traits with vastly different developmental origins or functional roles. Previous studies in fishes have demonstrated how shape covariation between traits is influenced by a combination of genetic, biomechanical, environmental, and developmental inputs (Conith et al., 2018; Evans et al., 2019; Larouche et al., 2018), and this covariation can manifest both locally (e.g., between the mandible and its associated adductor musculature) and distantly (e.g., between the mandible and the atrium). Local (i.e., among adjacent structures) covariation may manifest as a product of biomechanical and/or developmental inputs as the structures must act together toward some shared functional goal, and likely use shared molecular signals (e.g., “organizers”) to coordinate patterns of growth (Anthwal et al., 2015; Conith et al., 2019; Rot‐Nikcevic et al., 2007). Distant (i.e., among nonadjacent structures) covariation, on the other hand, may arise due to genetic effects such as pleiotropy, developmental effects such as cellular migration, and biomechanical effects such as shared functional roles between multiple structures involved in feeding and locomotion (Conith & Albertson, 2021; Conith et al., 2021; Hallgrímsson et al., 2009; Sánchez‐Villagra et al., 2016; Young et al., 2005).

**Figure 1:**
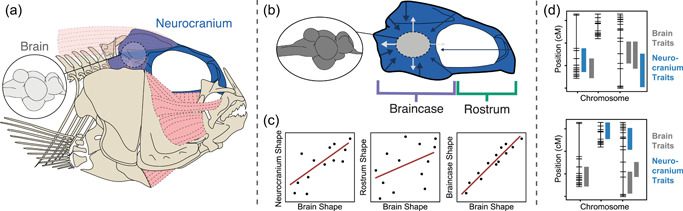
Cichlid brain–neurocranium anatomy and the implications for covariation. (a) Line drawing demonstrating the relative positions of the brain (gray) positioned within the braincase region of the neurocranium (blue). (b) The two major subregions of the neurocranium are the rostrum (anterior) and braincase (posterior). These regions were developmentally and statistically defined based on tissue origin and modularity analyses (see Materials and methods/Results section), respectively. (c) Hypotheses for the phenotypic association between the brain and neurocranium, alongside its subregions. Given the brain likely acts as an “organizer” of craniofacial shape, each neurocranial subregion will receive a different concentration of morphogens diffusing from the brain (braincase, high; rostrum, low), suggesting proximity of the brain to a given tissue should manifest in the strength of covariation between structures. (d) Hypotheses for the genetic association between the brain and neurocranium. Top, the genetic map depicts substantial overlap between brain and neurocranium traits, suggesting similar genomic regions may regulate both structures, consistent with pleiotropy. Bottom, the genetic map depicts little overlap between brain and neurocranium traits, suggesting different genomic regions may regulate each structure, consistent with processes above the level of genome regulating covariation. [Color figure can be viewed at wileyonlinelibrary.com]

The brain and the neurocranium provide an example of how two structures with very different genetic underpinnings, developmental origins, and functional roles must grow in unison (Figure 1a). Synergy between the brain and the neurocranium is critical to “normal” craniofacial development (Hu et al., 2015; Marchini et al., 2021; Richtsmeier & Flaherty, 2013), without which numerous clinical phenotypes can arise, such as holoprosencephaly, craniosynostosis, anencephaly, Down syndrome, and so on (Llambrich et al., 2022; Marcucio et al., 2011; T. E. Parsons et al., 2011). Slight differences in the timing (i.e., heterochrony) or position (i.e., heterotopy) of gene expression during brain and neurocranium development can generate stark differences in the resultant phenotype (Hu & Marcucio, 2009; Koyabu et al., 2014). In fishes, the neurocranium is derived from the mesoderm and neural crest cells (Kague et al., 2012), with most of the rostrum derived from the neural crest, while much of the braincase is derived from the mesoderm (Figure 1b). The brain forms from the neural ectoderm, developing into the neural tube before becoming more differentiated and forming the five major regions of the brain (telencephalon, diencephalon, mesencephalon, metencephalon, and myelencephalon) during early embryogenesis (Rocha et al., 2020). During this time, the brain appears to perform as an “organizer” in vertebrate cranial development, acting to determine the position of neurocranial sutures, directing cranial vault bone growth, and dynamically responding to mechanical stimuli during development (Marchini et al., 2021). Elucidating how this coordination proceeds (i.e., via pleiotropy, development, etc.) has proved difficult due to the complex nature of studying brain and neurocranium anatomy through ontogeny (Mork & Crump, 2015). Understanding this crosstalk between the brain and the neurocranium would provide insights into many clinical and evolutionary questions, including a better understanding of cranial dysmorphologies with different etiologies, and how selection can guide cranial evolution.

Here, we characterize the degree of association between the neurocranium and the brain to examine if potential morphological covariation stems from genetic covariation (e.g., pleiotropy). We utilize a hybrid mapping cross between two closely related rock dwelling (*mbuna*) cichlids from Lake Malawi that differ in their foraging behavior and extract three‐dimensional (3D) shape information from the brain and the neurocranium. We begin by characterizing the shape of the neurocranium and the brain and examine covariation between these structures, before examining the degree of covariation between the brain and two modules of the neurocranium: the braincase and rostrum. Dividing the neurocranium into two modules will determine the extent to which brain shape covaries with the shape of a local structure (i.e., the braincase) and a distant structure (i.e., the rostrum), and if the strength of this covariation bears out these differences in proximity to the brain (Figure 1c). We then investigate the genotype–phenotype (G–P) map using quantitative trait loci (QTL) analysis, to examine which regions of the genome underlie covariation in brain and neurocranium morphology. If pleiotropy underlies the covariation between the brain and neurocranium traits, we expect there to be overlap between QTL intervals (Figure 1d, top). Alternatively, the lack of genomic overlap between the brain and neurocraium traits would indicate that developmental interactions occurring at a level above the genotype are driving covariation (Figure 1d, bottom). Together, these results will help to parse the factors that contribute to craniofacial development across disparate tissue types and establish a foundation upon which to investigate the molecular mechanisms that regulate these dynamic processes.

## 2 MATERIALS AND METHODS

### 2.1 Generating a hybrid cichlid population

*Labeotropheus fuelleborni* (LF) and *Tropheops* sp. “red cheek” (TRC) are two closely related cichlid species that reside in shallow, rocky environments and feed by plucking attached algae from rocks (Ribbink et al., 1983). These two cichlids exhibit well‐documented differences in the craniofacial anatomy (Albertson, 2008; Conith & Albertson, 2021; K. J. Parsons et al., 2014), which reflect divergence in feeding behavior and microhabitat (Lloyd et al., 2021). LF is a specialized algae scraper with a relatively short and robust craniofacial anatomy, relatively small eyes, and a diurnal activity pattern. TRC is also a benthic forager, but feeds in a different manner than LF, utilizing a nip‐and‐twist mode of detaching algae from the substrate. In addition, TRC will more readily use alternate modes of foraging such as sifting and suction feeding. Accordingly, this species possesses longer heads and narrow jaws (Albertson & Pauers, 2019; K. J. Parsons et al., 2014). Finally, TRC has relatively large eyes, and a nocturnal activity pattern (Lloyd et al., 2021).

To create a hybrid population of cichlids for genetic mapping we crossed a wild‐caught TRC male from Chizumulu Island with a wild‐caught LF female from Makanjila Point. The full‐sibling F_1_ population was then intercrossed by randomly interbreeding individuals from different families for multiple generations until we obtained an F_5_ hybrid population (*n* = 636). The F_5_ hybrid population used in this study utilized 159 individuals reared in 40‐gallon glass aquaria on a 14 h light/10 h dark daily cycle in a recirculating system. All cichlids were fed a diet of algae and egg yolk flakes, limiting environmental variation. Hybrid animals were collected at ~5 months for genetic and phenotypic analysis. All animal experiments, husbandry, and housing were conducted in compliance with the Institutional Animal Care and Use Committee (IACUC) at UMass Amherst (#2018‐0094 to R. C. A.).

### 2.2 X‐ray scanning

We subjected our F_5_ hybrid population to x‐ray micro computed‐tomography (µCT) scanning to obtain 3D information on the neurocranium and brain shape. All hybrids were scanned twice: first to obtain hard‐tissue morphology (i.e., skeletal tissues), and second to gain soft‐tissue morphology following contrast‐enhanced staining protocols (i.e., neural tissues). We performed all µCT scanning using an X‐Tek HMXST 225 (Nikon Corporation). Hard‐tissue skeletal scans were acquired at 25–35 µm resolution using 95 kV and 90 µA. We captured soft neural tissue morphology by submerging our hybrid cichlids in 2.5% Lugol's iodine solution for 24 h. Lugol's iodine is radiopaque, permitting the visualization of the brain via µCT scanning once the solution has fully penetrated neural tissues (Hedrick et al., 2018). All iodine‐stained specimens were scanned at 20–25 µm resolution at 115 kV and 105 µA with a 0.1 mm copper filter. We then segmented the hard and soft tissues using Mimics (v.19 Materialise NV) and exported the 3D models to Geomagic (v.1.0 3D Systems) to remove noise (i.e., small floating voxels) by filtering for small, disconnected components before morphometric analysis.

### 2.3 Morphological analysis

**Figure 2:**
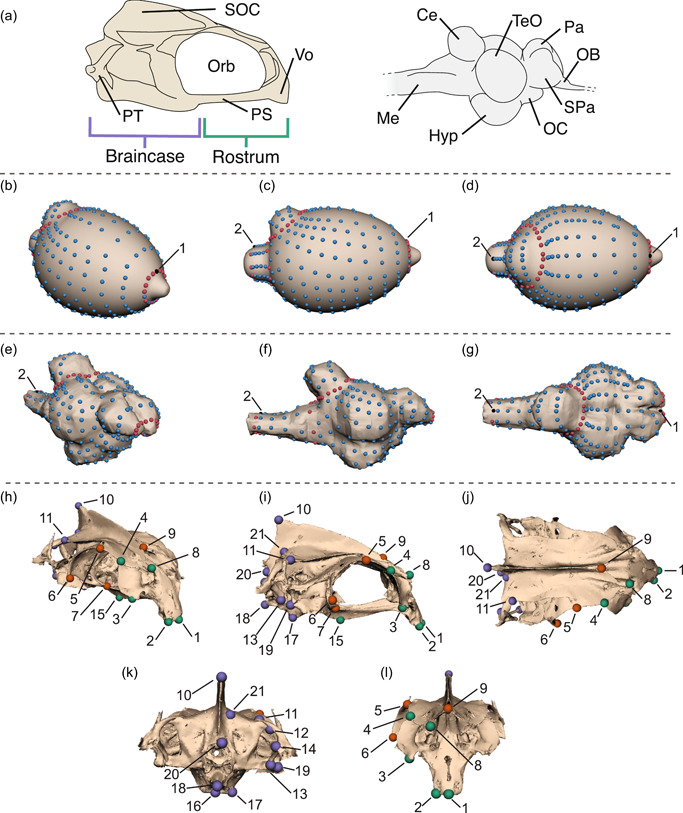
Brain and neurocranium anatomical outlines and landmark placement. (a) Left, cichlid neurocranium depicting the major subregions, the braincase and rostrum. Right, cichlid brain depicting the major subregions discussed in this study. (b–d) CAD templates used for automated mapping of surface semilandmarks onto brain models. (e–g) Fixed (black) and sliding semilandmarks (red) placed on the template match those placed on all hybrid brain models to assist in directing automated surface sliding semilandmark locations (blue). (h–j) Neurocranium fixed landmark placement positions across all hybrid cichlid individuals. Landmark color scheme reflects module partitions: green, rostrum module; red, shared rostrum/braincase module; purple, braincase module. (b–j) From left to right: left ¾ view, lateral view, dorsal view. Detailed information on landmark placement can be found in Table 1. Anatomical naming conventions taken from Liem and Osse (1975). Neurocranium: Orb, orbit; PS, parasphenoid; PT, posttemporal SOC, supraoccipital crest; Vo, vomer. Brain: Ce, cerebellum; Hyp, hypothalamus; Me, medulla; OB, olfactory bulbs; OC, optic chiasm; Pa, pallium; Spa, subpallium; TeO, optic tectum. [Color figure can be viewed at wileyonlinelibrary.com]

We characterized the shape of the neurocranium and brain from μCT‐scanned specimens using 3D geometric morphometrics (Figure 2 and Table 1). We characterized the shape of the brain using a combined human and semiautomated landmarking procedure, following recommendations and scripts outlined by Felice and Goswami (2018), whereby fixed landmark placement is governed by homology and semilandmark placement acts as a guide for the automated placement of the sliding surface semilandmarks. We began by designing a simplistic brain‐like model template using computer‐aided design (CAD) software (FreeCAD v.0.16.6712). This model broadly mimicked the major regions of the brain (Figure 2b–d). We placed two fixed LMs and 55 sliding semilandmarks from four separate curves on the CAD model. We then placed 297 surface semilandmarks across the CAD model to generate a high‐resolution anatomical characterization of the brain template. Finally, we placed the fixed and sliding semilandmarks onto our segmented brain scans in positions that corresponded to their position in the CAD model. The location of the fixed and sliding semilandmarks acted as a guide to help direct the semiautomated placement of the 297 surface semilandmarks across our segmented hybrid brains (Figure 2e–g). We used the R package *Morpho* to map the surface semilandmarks from the CAD model to all brain specimens using the placePatch function (Schlager, 2017, 2018). For the neurocranium, we placed 21 fixed landmarks at key functional and developmental positions (i.e., processes, muscle insertion points, sutures, etc.) across the braincase and rostrum (Figure 2h–l and Table 1). All nonautomated landmark placement was performed using Landmark Editor (Wiley et al., 2005).

**Table 1:** Description of landmark positioning for geometric morphometric analyses

Neurocranium landmarks		
Landmark number	Module	Description
1	Rostrum	Anterior–ventral tip of the vomer
2	Rostrum	Anterior–lateral tip of the vomer
3	Rostrum	Anterior–ventral tip of the lacrimal
4	Rostrum	Posterior connection between the lacrimal and frontal
5	Rostrum/braincase	Dorsal apex of the orbit
6	Rostrum/braincase	Posterior–ventral tip of the orbit
7	Rostrum/braincase	Lateral size of the prootic–parasphenoid suture
8	Rostrum	Anterior tip of the frontal lateral line canal
9	Rostrum/braincase	Anterior tip of the supraoccipital crest
10	Braincase	Posterior–dorsal tip of the supraoccipital crest
11	Braincase	Dorsal–anterior tip of the parietal–posttemporal suture
12	Braincase	Vental–posterior tip of the parietal–posttemporal suture
13	Braincase	Vental tip of post temporal–exoccipital suture
14	Braincase	Posterior region of the posttemporal
15	Rostrum	Posterior–ventral apex of the parasphenoid
16	Braincase	Midline of the synovial joint of the upper pharyngeal jaw
17	Braincase	Lateral edge of the upper pharyngeal jaw—synovial joint
18	Braincase	Posterior–ventral tip of the basiooccipital
19	Braincase	Posterior–lateral edge of the exoccipital
20	Braincase	Dorsal tip of the foramen magnum
21	Braincase	Base of the supraoccipital crest on the epiotic

Brain landmarks		
Landmark number	Type	Description
1	Fixed	Anterior tip of the brain
2	Fixed	Posterior tip of the brain
3–17	Semi	Olfactory bulb demarcation
18–42	Semi	Cerebellum demarcation
43–50	Semi	Anterior portion of the medulla
51–57	Semi	Posterior portion of the medulla
58–354	Surface	All surface landmarks

*Note*: Numbering scheme matches that depicted in Figure 2. Top, landmark descriptions for the neurocranium analysis, and includes module partition information. Bottom, landmark descriptions for the brain analysis, which includes notes on which were fixed, semi‐, or surface landmarks.

Once all hybrid neurocrania and brains were digitized, we used general least‐squares Procrustes superimposition (GPA) to remove the effects of size, translation, and rotation from the landmark configurations of all our specimens with the gpagen *geomorph* function (Adams et al., 2021; Baken et al., 2021; Rohlf, 1998). To examine potential allometric effects in our neurocranium and brain data, in each case, we performed a multivariate regression of shape on centroid size using the procD.lm function in *geomorph* (Collyer & Adams, 2018). We found a significant effect of size in our shape data for both our structures (neurocranium, *r* ^2^ = 0.047, *Z* = 5.62, *p* = .001; brain, *r* ^2^ = 0.032, *Z* = 3.16, *p* = .001). We, therefore, removed the allometric component of shape variation by extracting neurocranium and brain landmark residuals from the multivariate regression for use in subsequent analyses.

The neurocranium is traditionally thought to be comprised of two primary subunits, or modules (Figure 2a), comprising the rostrum, which houses the eyes and provides numerous origin sites for the feeding musculature, and the braincase, which houses the brain and supports the epaxial musculature via the supraoccipital crest (Evans et al., 2017). We statistically confirmed the presence of these modules by partitioning our landmarks into either a braincase or rostrum module and examining the degree of integration between each module (Figure 2b, braincase partition indicated by purple landmarks and rostrum partition indicated by green/red landmarks). We explicitly quantified modularity using the covariance ratio (CR) coefficient (Adams, 2016). To obtain the CR coefficient, we first assessed pairwise covariances between landmarks within modules and then assessed pairwise covariances between landmarks among modules, and calculated a ratio from the between‐ versus among‐landmark covariances. For significance testing, this observed CR coefficient is compared to a distribution of simulated CR coefficients generated by randomly assigning landmarks to either module 10,000 times. Modularity analyses were performed using the modularity.test function in the R package geomorph.

**Figure 3:**
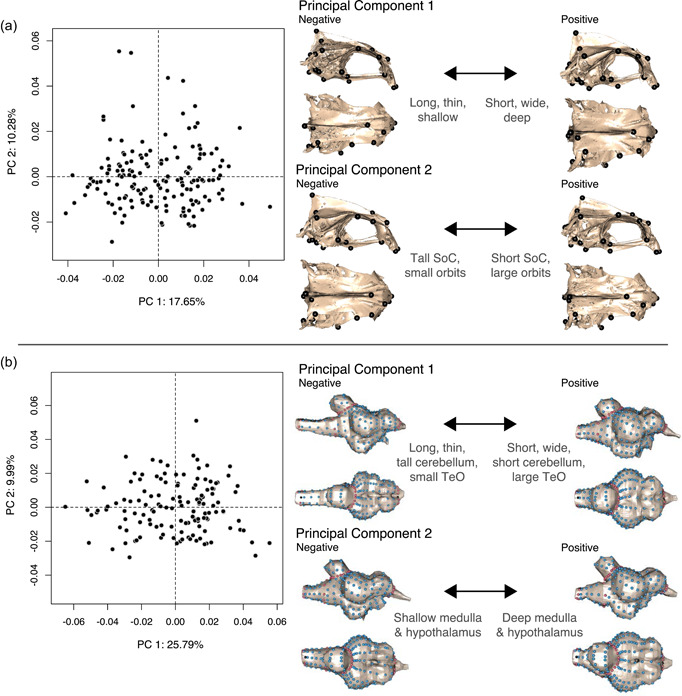
Neurocranium and brain principal component morphospaces for all F_5_ hybrid individuals. Primary and secondary axes of variation with representative individuals illustrate morphological differences among the hybrid population. Representative neurocranium and brain images are presented in the right lateral (top) and dorsal (bottom) views for all structures. (a) Neurocranium morphospace and representative individuals at the terminal ends of each axis. (b) brain morphospace and representative individuals at the terminal ends of each axis. [Color figure can be viewed at wileyonlinelibrary.com]

We found strong evidence for braincase and rostrum modules in our hybrid neurocrania (CR = 0.854, *Z* = −3.06, *p* = .0002). Despite this result, our braincase landmarks did not capture the length of the braincase, and, as a result, we decided to test another modularity hypothesis that would enable the central region of the neurocranium to vary (Figure 2). This three‐module hypothesis also gained significant support (CR = 0.866, *Z* = −1.86, *p* = .0324). While both hypotheses provide significant evidence for modularity between the braincase and rostrum, we compared the effects sizes of each hypothesis to examine if the two‐ or three‐module hypothesis had statistically greater support. Using the compare.CR function from the R package geomorph, we observed no statistical difference between hypotheses, indicating both modularity hypotheses are equally likely (pairwise difference in CR effect size = 0.578, *p* = .563). As a result, we decided to split the neurocranium into two primary regions (braincase and rostrum) that shared the central complement of landmarks for subsequent analyses. Including these shared landmarks allowed us to more accurately characterize the length aspect of the brain and neurocranium shape (Figure 2, red landmarks are shared between configurations). Finally, we performed a principal component (PC) analysis on the neurocranium and brain landmark data, and plotted the first two PC axes to observe the major differences in shape variation among specimens (Figure 3).

### 2.4 Shape covariation

We assessed the degree of covariation among our hybrid neurocranium and brain Procrustes‐aligned landmark configurations using two‐block partial least‐squares analysis. Partial least‐squares (PLS) analysis examines all the dimensions of a highly multivariate trait (i.e., neurocranium and brain shape) to find axes pertaining to maximum covariation between shapes (Adams & Collyer, 2016; Rohlf & Corti, 2000). Given the modular nature of the neurocranium, we also examined the strength of covariation between the brain and the rostrum and the brain and the braincase, predicting that if there is strong developmental feedback between the hard and soft tissues, we should expect to observe the tightest correlations between the brain and braincase. We used the two.b.pls *geomorph* function in R to conduct all PLS analyses. Finally, we compared the effect sizes of each PLS analysis to statistically compare differences in the strengths of covariation among the brain and the rostrum/braincase. We used the compare.pls function from the R package *geomorph* to assess pairwise differences in effect sizes.

We extracted the *x*–*y* coordinates from the resultant two‐block PLS analysis. PLS scores extracted from this analysis reflected the primary axes of covariation *between* a pair of measured traits (as opposed to a PC analysis where the shape scores reflect the primary axes of variation *within* a trait). Each “trait” can therefore be thought of as reflecting a primary trait (i.e., the trait represented by that PLS block), and a covarying trait (i.e., the trait that the primary trait is regressed against). However, when this trait is mapped using a QTL analysis, the trait “complex” is treated as a single variable. The main reason we wanted to use the major axes of *covariation* regards the utility of QTL analysis to assess multivariate traits. There are currently no methods that allow us to perform QTL analysis using multivariate data. Our only option is to split up our traits and run each independently. Therefore, the brain trait will be assessed three times as the covarying hard‐tissue trait is cycled through (e.g., 1, brain–neurocranium; 2, brain–rostrum; 3, brain–braincase). While not an unbiased approach, this analysis allowed us to more explicitly compare covariation at the phenotypic and genetic levels. The resultant map will retain consistency between those traits examined in the integration analyses, and what is assessed in the statistical genetics analyses (Conith et al., 2021).

### 2.5 QTL mapping

Using an F_5_ population allowed for the accumulation of recombination events with each successive generation, increasing the resolution of mapping intervals ready for genotyping and the production of a genetic map (Albertson et al., 2014). However, performing additional intercrosses following the F_2_ generation resulted in our F_5_ hybrid population diverging from a more tractable pattern of Mendelian inheritance, complicating map construction. As a result, we used the original F_2_ genetic map of the same pedigree and restriction site‐associated DNA (RAD) genotyping to provide a foundation for the F_5_ map (Albertson et al., 2014). We then genotyped a subset of genetic markers across the genome (*n* = 812) in the F_5_ population using the same RAD‐seq methodology used in the F_2_ to produce our final map for QTL analysis. See Albertson et al. (2014) and Conith et al. (2021) for full details of map construction.

We performed a QTL analysis using the primary axes of covariation between a pair of traits extracted from the two‐block PLS analysis. Each block was run through the QTL analysis to pinpoint potential genomic regions that reflected an association between phenotype and genotype. We searched for putative loci using the multiple QTL mapping (MQM) method (Broman & Sen, 2009) implemented in r/qtl (Broman & Wu, 2020; Broman et al., 2003). We started by using the scanone function to conduct an initial QTL scan. We then sequentially added cofactors to the model and used maximum‐likelihood backward elimination with the mqmscan function to determine the fit of each cofactor. We progressively added more cofactors to the model until we maximized the logarithm of odds (LOD) score. We assessed QTL marker significance using the mqmpermutation function at the *α* = .05 and *α* = .1 levels. The mqmpermutation function disassociates the relationship between genotype and phenotype by shuffling the phenotypic data relative to genotypic data 1000 times, to generate a null distribution (Arends et al., 2010). We considered a QTL marker to be significant if the LOD scores exceeded the *α* = .05 LOD threshold level. Following the identification of a significant QTL peak, we calculated an approximate Bayesian credible interval in which a potential candidate locus would reside within using the function bayesint.

In those instances where we observed overlap among the Bayesian credible intervals of brain and neurocranium traits, we performed explicit tests for pleiotropy. Based on the output from our QTL analysis, there were two covarying traits (brain and braincase) that exhibited overlapping Bayesian credible intervals on LG 7. There were no other regions with overlapping intervals between hard and soft tissue covarying traits. We therefore quantitatively determined if the brain/braincase intervals can be considered pleiotropic or are associated with separate QTL. To determine whether a QTL can be considered pleiotropic, we used a likelihood‐ratio test (LLRT) that compares the likelihood of the two traits exhibiting a QTL that is common to each trait, or distinct (Jiang & Zeng, 1995). The test statistic is calculated as the logarithm of the ratio between these two likelihood values. Significance testing of pleiotropy versus separate QTL was obtained via parametric bootstrapping (Boehm et al., 2019). A significant result indicates the two traits localize to a distinct QTL (i.e., *p* < .05), while a nonsignificant result indicates the two traits localize to a common QTL (i.e., *p* > .05). We performed the pleiotropy and bootstrapping tests using the scan_pvl function from the qtl2pleio package (v1.4.3) in R (Broman et al., 2019).

We used a fine‐mapping approach to further investigate the genotype–phenotype relationship in greater detail along LG 7. We identified additional RAD‐seq single‐nucleotide polymorphism (SNPs) in the F_5_ population and used the Maylandia zebra genome to anchor QTL intervals to particular stretches of physical sequence along LG7, gaining a marker every ~490 kb.

Following the completion of the LG 7 fine map, we then merged our brain and braincase covariation data with the map and calculated the difference on average trait values between F_5_ hybrids with the LF genotype and hybrids with the TRC genotype. We calculated the difference between genotypes at every marker in the LG 7 map using the effectsplot function in r/qtl. We then assessed genetic divergence between wild‐caught LF (*n* = 20) and TRC (*n* = 20) populations within the Bayesian credible interval on LG 7 (~40–~53 Mbs) to find markers that exhibited a combination of high phenotypic effects and alternate fixation of alleles between populations (Albertson et al., 2014). To this end, we calculated *F* statistic estimates (*F*_ST_) at each loci (Nei, 1987). *F*_ST_ values close to 1.0 indicate high differentiation among populations at a given loci.

## 3 RESULTS

### 3.1 Shape variation in the neurocranium and brain

By crossing LF with TRC, we gained a range of craniofacial morphologies that encompassed the full spectrum of phenotypes between these two species, as well as transgressive phenotypes. In the neurocranium, morphological variation reflected differences among the parental species (Figure 3a), including short, wide, and deep neurocrania similar to what is seen in the specialized algivore LF and relatively long, thin, shallow neurocrania similar to the more generalist algivore TRC. Many other aspects of morphological variation in the F_5_ have implications for the ability of an individual cichlid fish to resist compressive and torsional forces, including the angle of the vomerine process and size/shape of the parasphenoid, which can impact the propagation of stress from the rostrum to the rest of the skull (Cooper et al., 2011).

Our PC morphospaces revealed how the neurocrania and brains varied among our hybrid population of cichlids (Figure 3). Neurocranium PC1 reflected broad differences in length, width, and depth (17.65%), while PC2 represented differences in the size of the supraoccipital crest and the orbits (10.28%). In assessing the brain PC space, we observed that PC1 accounted for changes in the length, width, and relative size of the cerebellum and optic tectum (25.79%), while PC2 reflected differences in the relative size of the hypothalamus and medulla (9.99%).

Variation among brain regions may reflect differences in foraging behavior and day/night activity patterns in the parental species (Figure 3b). Previous work has demonstrated that TRC has relatively larger eyes compared with LF (Lloyd et al., 2021). If TRC are visually biased nocturnal foragers, then large eyes could likely impact the relative size of the optic tectum, a brain structure that we found exhibited a high degree of variation among our hybrids. We also observed differences in the relative size of the hypothalamus, which is involved in a number of different social, aggressive, and sleeping behaviors (Loomis et al., 2019). Cerebellum and medulla shapes also varied among hybrids, structures that are involved in various motor functions, and play roles in respiratory and circulatory control (Hirsch et al., 2021). We found little variation in the telencephala and olfactory bulbs among hybrids, possibly due to the similarity in their algal scraping/nipping diets (Sylvester et al., 2010).

### 3.2 Shape correlations between the neurocranium and brain

**Figure 4:**
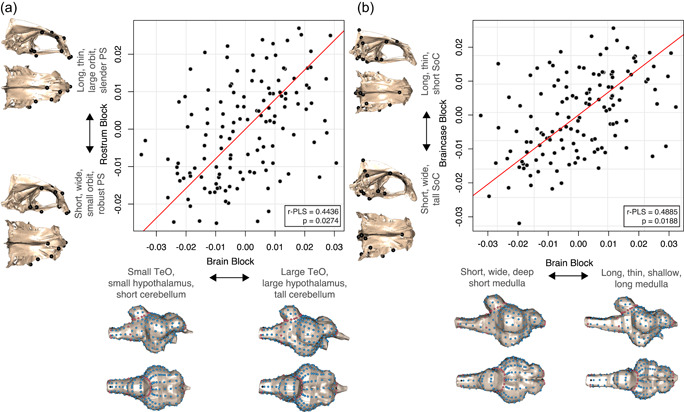
Two‐block partial least‐squares analysis to assess the association between the brain and the constituent parts of the neurocranium: the rostrum and braincase. Representative hybrid individuals are included to illustrate the morphological differences across the primary axes of covariation. (a) Brain and rostrum. (b) Brain and braincase. [Color figure can be viewed at wileyonlinelibrary.com]

We observed an association between the neurocranium and brain (r‐PLS = 0.492, *Z* = 1.905, *p* = .0255; Supporting Information: Figure S1), and even when we partitioned the neurocranium into rostrum and braincase modules, significant associations remained (Figure 4b,c and Supporting Information: Figure S2a–c). Notably, differences in the strength of shape covariation reflected the proximity of tissues to one another. Specifically, the association between the rostrum and brain (r‐PLS = 0.444, *Z* = 1.898, *p* = .0274; Figure 4a) was weaker than the association between the braincase and brain (r‐PLS = 0.489, *Z* = 2.072, *p* = .0188; Figure 4b). However, when we directly compared the effect sizes between each PLS analysis, the strength of covariation between the brain and rostrum/brain and braincase was statistically equivalent (pairwise effect sizes = 0.156, *p* = .876). Similarly, when investigating each module independently (i.e., no landmarks shared between the rostrum and braincase configurations), we still found evidence for associations between the rostrum and brain (r‐PLS = 0.511, *Z* = 2.214, *p* = .0068), and the braincase and brain (r‐PLS = 0.541, *Z* = 3.244, *p* = .0008). Similar to the two‐module hypothesis, when we directly compared the effect sizes between each PLS analysis for the three‐module hypothesis, we found that the strength of covariation between the brain and the neurocranium modules was statistically equivalent (Supporting Information: Table S1). These trends in the strength of association between the brain and the different modules of the neurocrainum lend support to our initial hypothesis that the brain acts as a central “organizer” of neurocranium shape.

While much of the association can be attributed to large‐scale covariation in length, width, and depth between the brain and neurocranium (Supporting Information: Figure S1), certain subregions of the brain also appear to covary with distinct aspects of the neurocranium. We observed a significant association between the brain and rostrum (Figure 4a), indicating that integration can arise between the brain and rostrum despite distinct functional demands and spatial positioning.

### 3.3 Genetic mapping provides evidence for genetic and developmental contributions to shape variation

**Figure 5:**
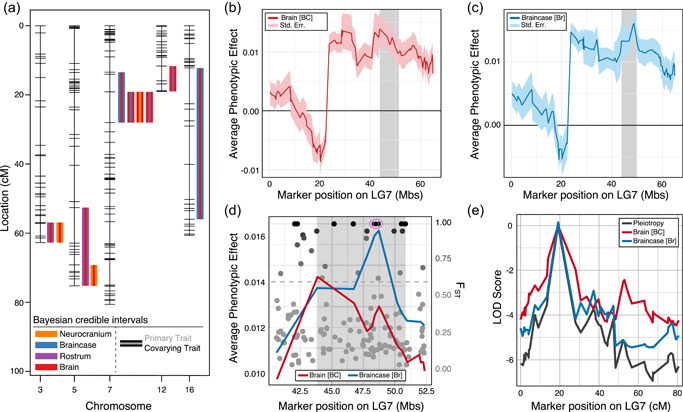
Statistical genetic analyses to uncover regions of the genome responsible covariation between brain and neurocranium. (a) Quantitative trait loci analysis to identify regions in the genome associated with each covarying trait. (b) Fine‐mapping brain–braincase covariation and (c) braincase–brain covariation across LG 7. The trait in square brackets in the figure legend represents the covarying trait. Values far from 0 denote large differences in trait values between hybrids with either a *Labeotropheus fuelleborni* (LF) or *Tropheops* sp. “red cheek” (TRC) genotype at a given marker. We observe peak genotype‐phenotype association at ~50 Mb that coincides with our Bayes credible interval (gray bar). Solid line represents the mean phenotypic effect and lighter polygon represents the standard error. (d) Fine mapping the brain–braincase covarying traits across the Bayes credible interval on LG 7. Population level genetic diversity (*F*_ST_) data are included on the map (black points) with their opacity dependent on the degree of marker segregation between LF and TRC. Within the credible interval (gray bar), there are three *F*_ST_ values of 1.0 (indicating alternate fixation) that reside close to a genotype–phenotype peak for both traits (purple circle). (e) Pleiotropy analysis across LG 7 to assess evidence of shared genetic control of brain and braincase trait covariation. The pleiotropy peak between traits occurs at approximately 20 centimorgans (cM) along LG 7. [Color figure can be viewed at wileyonlinelibrary.com]

From our QTL analysis, we pinpointed multiple regions in the genome that underlie covariation in brain and neurocranium shape (Figure 5a). Since shape data for mapping were extracted from the PLS analysis, when describing the traits we list the primary trait first and the covarying trait in square brackets. We found a single significant QTL (at the <0.05 genome‐wide level) for the neurocranium [brain] and two for the brain [neurocranium]. When we partitioned out the neurocranium landmarks into two modules, we recorded two significant QTL for the rostrum [brain] alongside two significant QTL for the brain [rostrum], and one for the braincase [brain] with two significant QTL for the brain [braincase]. In a single case, QTL did not overlap between the neurocranium and the rostrum (LG 12). This indicated that some axes of covariation specific to the rostrum [brain] were not initially realized when using the full complement of neurocranial landmarks, permitting greater resolution in the genotype–phenotype map to identify exactly what regions may shape rostrum covariation.

All significant QTLs explained between 7% and 22% of the covariation in the F_5_ population, and modes of inheritance included additive, dominant, and overdominant effects. We identified significant LOD scores on 5 of 25 linkage groups (LG); however, some LGs clearly represented QTL hotspots where we observed substantial overlap among multiple traits. In particular, LGs 3, 5, and 7 reflected regions in the genome where QTL overlapped from multiple covarying hard tissue traits. However, only LG 7 reflected a region with QTL overlap between the hard‐ and soft‐tissue traits.

The brain [braincase] and braincase [brain] traits overlapped across two markers on LG 7, raising the possibility that covariation between traits was driven by a similar gene or genetic region. The fine mapping results showed peaks within the Bayesian credible intervals (Figure 5b,c), and were especially notable in the braincase [brain] trait. Additionally, the trace of the two traits tracked in a very similar fashion across the whole LG. When we examined the genomic region surrounding the Bayes credible interval, we observed a number of phenotypic effect peaks in both traits (Figure 5d). However, there was a region between 47.5 and 50 Mbs that combined high phenotypic effects with fully segregated *F*_ST_ markers (highlighted in purple in Figure 5d), indicating that (1) LF and TRC populations exhibited alternative SNPs at those markers and (2) F_5_ animals with alternate genotypes at that interval had different morphologies. Three *F*_ST_ markers with values of 1.0 surrounded the high phenotypic effect peak (Figure 5d). This region contains *notch1a*, a receptor that regulates cell differentiation and function and is known to be critical for regulating a host of processes during craniofacial development (Pakvasa et al., 2021). The upstream marker is ~60 kb from the promoter region of *notch1a*, while two downstream markers reside ~50 and ~260 kb from the 3′ end of *notch1a*.

We then explicitly tested for pleiotropy between the brain–braincase covariation traits on LG 7 as this was the only region where we observed overlap between a hard‐tissue neurocranium trait and a soft‐tissue brain trait, and we found strong evidence for shared genetic architecture (Figure 5e). Following an LLRT, we found that the overlap on LG 7 was nonsignificant between the brain and braincase (LLRT = 0.14, *p* = .54), indicating that we can accept the null hypothesis (no pleiotropy) and conclude that this interval likely contains a pleiotropic locus. No traits derived from the covariation analyses between the brain and rostrum, nor the brain and neurocranium resulted in overlapping QTL.

## 4 DISCUSSION

### 4.1 Roles for genetics and development in shaping brain and neurocrainum covariation

The vertebrate head is a multifunctional structure that is involved in a myriad of tasks, each with competing demands, but the range of possible head morphologies is bound by genetic, developmental, and functional constraints that selection must navigate (Hallgrimsson et al., 2019). Here, we document shape associations between the brain and neurocranium and find evidence for pleiotropic control of this shape covariation. Using 3D geometric morphometrics to characterize shape via high‐resolution landmark configurations permits a more holistic understanding of those specific morphological aspects of the brain and neurocranium that appear correlated and the same suite of genes may regulate this covariation.

We observed the strongest association between the brain and braincase, the two structures in which feedback between each should be high given their close proximity (Figure 4b). We found differences in the length, width, and depth of the braincase tracked with differences in length, width, and depth of the brain. While much of the variation between brains in the length dimension appears to be biased around the medulla, differences in depth appear to demonstrate an equal contribution from the hypothalamus, optic tectum, and cerebellum, in that all regions appear to covary in unison. We also found explicit evidence for pleiotropy between these covarying traits. The region we identified on LG 7 was previously implicated in oral and pharyngeal jaw bone shape variation and is home to a handful of notable candidate genes, including *smad7*, *dymeclin*, and *notch1a* (Conith & Albertson, 2021). The braincase has a peak genotype–phenotype association at *notch1a*, which encodes a receptor for the Notch signaling pathway, is known to be crucial for craniofacial embryogenesis and can impact a wide variety of tissues, from skeletal to muscular and neural (Ables et al., 2011; Pakvasa et al., 2021). Previous studies have demonstrated differences in *notch1a* expression between LF and TRC in two boney craniofacial structures, the lower oral and pharyngeal jaw (Conith & Albertson, 2021). LF exhibited low *notch1a* expression levels with low variation in expression, while TRC exhibited high expression levels with high variation in expression. Higher expression of *notch1* appears to inhibit osteoblast differentiation and mineralization by reducing Wnt/β‐catenin signaling (Ballhause et al., 2021). Osteoblasts serve to build new bone during development, and in impairing their differentiation, *notch1* could reduce bone formation to produce more slender bones (Zanotti & Canalis, 2016), features that are characteristic of TRC. In situ hybridization for *notch1a* in zebrafish has revealed concentrations of expression across the head, and can be clearly seen in both the skeletal neurocranial tissues and the brain tissues (Chen et al., 2012, 2015; Kumar et al., 2017). Mutations in *notch1a* are also known to affect swimming behavior, directly impacting escape response movement (Liu et al., 2003). Taken together, *notch1a* represents a strong candidate gene to examine further and could represent a means by which brain and skull tissues coevolve, while simultaneously impacting foraging and swimming behavior.

The phenotypic association we observed between the neurocranium and brain provides a means to simultaneously alter behavior and craniofacial morphology. While associations are present between the brain and neurocranium, plenty of variation is left unaccounted for. Some unaccounted variation may be due to plasticity or variation due to developmental noise, and this may explain the lack of correlations at the genetic level. Indeed, despite phenotypic correlations in each assessment, we observed only a single instance of genetic correlation based on our QTL analysis (Figure 5a). The discrepancy between phenotypic and genetic associations among traits may result from developmental interactions through ontogeny (Conith et al., 2021), which can obfuscate the genotype–phenotype map and limit our ability to detect the genetic regions responsible for phenotypic covariation.

### 4.2 The brain as a regulator of neurocranium development

The brain appears to act as an important organizer of head development, with changes in neural anatomy arising over evolutionary time as populations adapt to their physical environment (Marchini et al., 2021; Marcucio et al., 2011; Teng et al., 2019). Initial hypotheses developed using mouse models theorized that developmental constraints produced a highly integrated brain with little variation among its constituent parts (Finlay & Darlington, 1995). However, recent studies using artificial selection and statistical genetics have determined that the brain develops as a mosaic, with subunits able to grow and develop relatively independently of each other (Barton & Harvey, 2000; Fong et al., 2022). This modularity in the relative growth rates across different subregions of the brain permits sensory biases to arise that are associated with specific ecological factors (Jaggard et al., 2020; Kozol et al., 2022). The presence of developmental modularity among brain regions may promote modularity across the skull, as well as impact the shape of the surrounding neurocranium, given the cross‐talk between both tissues (Fabbri et al., 2017; Marcucio et al., 2011). A direct link can therefore be drawn between behavior, which is determined by the relative size and shape of subregions across the brain, and neurocranium shape, which will differentially respond to the morphogenetic signals produced by the brain depending on the relative size and shape of those subregions.

In our F_5_ hybrids, differences in rostrum length, width, orbit size, and parasphenoid shape covary with the size of the optic tectum, hypothalamus, and cerebellum in the brain. We find that orbit size covaries with the size of the optic tectum, demonstrating a clear relationship between the size of the eye and the ability of the brain to process visual information. This is a common trend in cichlids, as the most visual hunters typically exhibit large optic tecta, the region of the brain responsible for processing visual stimuli, while those with scent based‐hunting exhibit large olfactory bulbs (Sylvester et al., 2010; York et al., 2019). The hypothalamus also appears to covary with the neurocranium, a trait we know may define differences among our parental species given the role of the hypothalamus in sleep and circadian rhythms (Lloyd et al., 2021). We, therefore, find evidence for both size and shape covariation between the brain and neurocranium, and while these measures can change in unison, it is also possible to decouple size from shape change, a feature that likely has far‐reaching effects on craniofacial development and behavior (Howell et al., 2021; Kozol et al., 2022).

We observed covariation among the brain and neurocranium among both local and distant structures. We found the strongest association between the brain and braincase (Figure 3c; rPLS = 0.489), a local association between two structures in close proximity. The braincase serves to house the brain and shares a similar developmental environment, but also has roles in providing the insertion sites for the epaxial musculature that are important in feeding and locomotion (Camp et al., 2015; Conith et al., 2019), although braincase shape is likely driven by brain morphology more so than a diet. Rostrum shape still exhibits a significant association with brain shape, but the strength of covariation is somewhat weaker than the braincase (Figure 4a; rPLS = 0.444). The rostrum has a different developmental origin (neural crest‐derived) relative to the braincase (mesodermally derived), and its proximity to the brain is more distant, which could weaken the effect of morphogens secreted by the brain given the lower concentrations (Hu et al., 2015). However, during early developmental stages, the brain resides in close proximity to tissues that will form the rostrum (i.e., ethmoid plate). As development proceeds, the brain shifts posteriorly. Forebrain transplantation experiments from duck to chick embryos have demonstrated the role of the anterior brain regions in early patterning of the rostrum and facial skeleton, producing facial shapes in chicks that exhibit duck traits (Hu et al., 2015; Marchini et al., 2021). It is therefore possible that the association between the rostrum and brain is due to interactions that occurred early in development. Additionally, the shape of the rostrum is likely to be more heavily impacted by functional demands governed by dietary ecology and foraging strategy, for example, the width, length, and angle of the vomer should reflect diet given that it articulates with the upper jaw apparatus (Cooper et al., 2011). Nevertheless, we still observe associations between the brain and rostrum, which speaks to the power of the brain as a major regulator of shape across the craniofacial skeleton and permits concomitant changes in behavior and diet (Tsuboi et al., 2014).

### 4.3 Brain and neurocranium covariation in evolution

Adaptive radiations, such as the cichlid fishes of Lake Malawi, are often characterized by phenotypic diversification along three major axes (Todd Streelman & Danley, 2003): habitat axes (i.e., deep‐shallow), trophic axes (i.e., hard/stationary prey–soft/evasive prey), and communication axes (i.e., territoriality, coloration). Diversification along the communication axes is common in Lake Malawi cichlids, as many taxa are territorial, exhibit vibrant coloration patterns, and even build bowers to attract mates (York et al., 2015). Additionally, behavioral evolution can occur rapidly, likely resulting in morphological changes to both the neurocranium and the brain (York et al., 2019). Many cichlids vary in their degree of territoriality, both within and between species, including the parentals used in this study to build the hybrid cross, with TRC being more territorial relative to LF (Ribbink et al., 1983). Given the centrality of behavioral modulation to cichlid life history, and how the transcriptional landscape within the brain can change markedly through ontogeny (Li et al., 2017), brain–neurocranium evolution could be fairly rapid.

Movement into vacant niches is a major mechanism that can promote speciation. These transitions into new environments often foster the evolution of novel behaviors that would likely trigger changes to brain anatomy (Aristide et al., 2016), and ultimately drive concomitant changes to the neurocranium (Eliason et al., 2021). Lake Malawi has experienced large variations in water level since it formed around two million years ago, producing a highly stochastic environment that forces the cichlids within the lake to rapidly shift between foraging habitats (Lyons et al., 2015). Cichlids, such as species within the *Tropheops* genus are known to rapidly and repeatedly transition between habitats in Lake Malawi, changing their trophic morphology in the process (Conith et al., 2020). The brain may play some role in assisting these transitions, especially given that we know brain morphology varies with ecology among cichlids (Sylvester et al., 2010). It is therefore tempting to speculate that feedback between the brain and skull has facilitated divergence across multiple axes of variation, thereby contributing to the evolutionary success of cichlids in Lake Malawi and across the East African Rift Valley.

## 5 CONCLUSIONS

The brain plays a central role as an “organizer” during craniofacial development (Marcucio et al., 2011). The tight developmental integration between the brain and neurocranium allows rapid, concomitant changes in both structures following behavioral shifts. These shifts may elicit the recruitment of neural tissues to specific subregions of the brain to accommodate such sensory biases, which could subsequently produce shifts in the location or concentrations of morphogens secreted from the brain to pattern the developing neurocranium. Our data demonstrate how this integration manifests at the phenotypic level, observing tight patterns of covariation between brain and neurocranium shapes. We find evidence for both pleiotropy and independent control of brain–neurocranium shape, indicating that a combination of genetic processes and developmental processes that act above the level of the genome (i.e., epigenetic processes, *sensu* Waddington, 1942) together contribute to covariation. An important aspect of this relationship is to understand how it changes over macroevolutionary timescales as modulating the strength of covariation between the brain and neurocranium could have far‐reaching effects for how rates of morphological evolution, morphological disparity, and evolvability manifest across a clade. Further, examining brain–neurocranium covariation in a phylogenetic context would provide an excellent model to understand how neuroanatomy can influence morphology and behavior. The outcomes of these studies can document the intricacies of brain and neurocranium coevolution, and more broadly how genetic and developmental processes can influence macroevolutionary processes (Eliason et al., 2021; Fabbri et al., 2017), an important question that is central to better understanding biodiversity.

## AUTHOR CONTRIBUTIONS

Andrew J. Conith and R. Craig Albertson designed research. Andrew J. Conith and Sylvie A. Hope collected data, performed research, and analyzed data. Andrew J. Conith and R. Craig Albertson interpreted data and wrote the manuscript. Andrew J. Conith, Sylvie A. Hope, and R. Craig Albertson edited the manuscript.

## Supporting information

Supporting information.

Supporting information.

Supporting information.

## Data Availability

All scripts and raw data used in this study can be found on GitHub, https://github.com/andrewjohnconith/BrainNeuroCoV.
