# Supplementary material for: Covariation of brain and skull shapes as a model to understand the role of crosstalk in development and evolution
Source: Evol Dev. 2022 Nov 14;25(1):85–102. doi: 10.1111/ede.12421 (PMC9839637; doi:10.1111/ede.12421)
Supplement: Supplementary file 1 — Supporting information. [file EDE-25-85-s001.pdf]

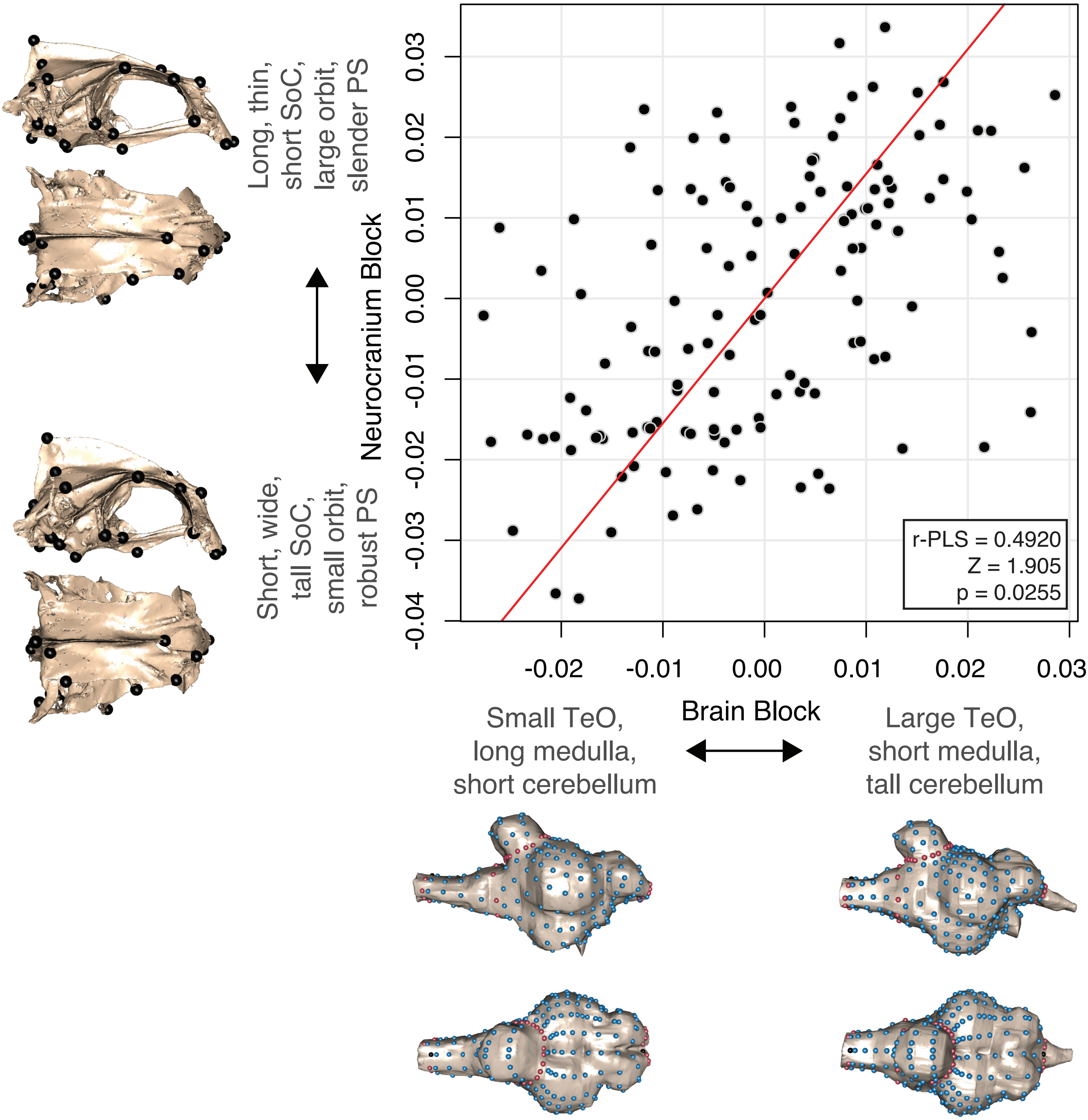

Figure S1. Two-block partial least squares analysis to assess association between the brain and the neurocranium. Representative neurocranian and brains from hybrid individuals are included to illustrate the morphological differences across the primary axes of covariation and landmarks used to characterize their shape are included. Statistical outputs for this assoication is listed in the legend. Neurocranium: black points, fixed landmarks. Brain: black points, fixed landmarks; red points, sliding semi-landmarks; blue points, sliding surface semi-landmarks.
