# Supplementary material for: Covariation of brain and skull shapes as a model to understand the role of crosstalk in development and evolution
Source: Evol Dev. 2022 Nov 14;25(1):85–102. doi: 10.1111/ede.12421 (PMC9839637; doi:10.1111/ede.12421)
Supplement: Supplementary file 2 — Supporting information. [file EDE-25-85-s003.pdf]

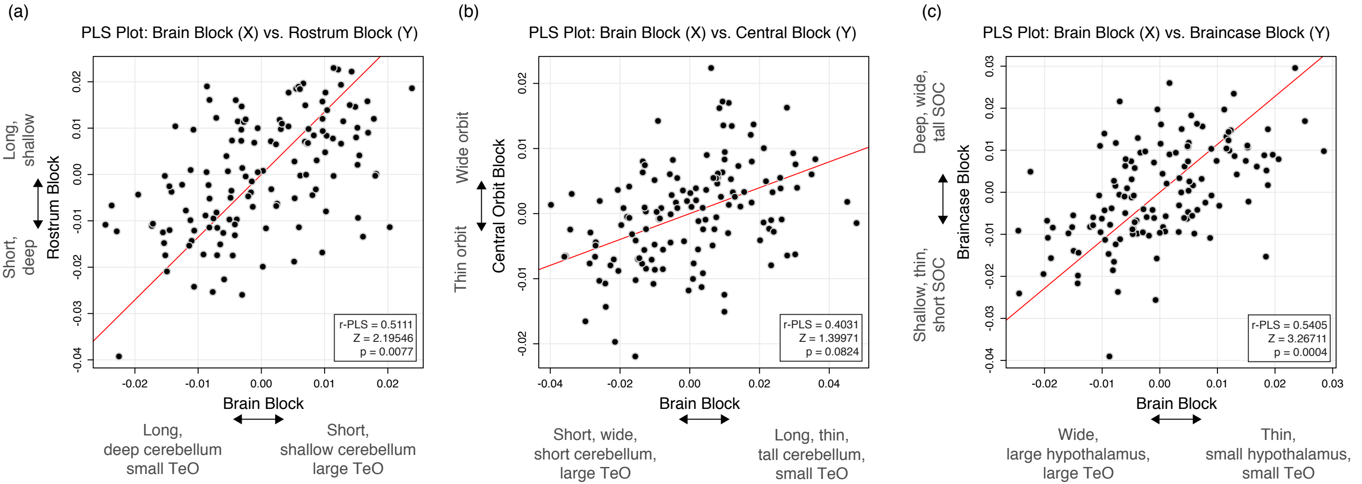

Figure S2. Two-block partial least squares analysis to assess association between the brain and the neurocranium and its constituent parts: the rostrum and braincase. Morphological differences across the primary axes of covariation for each anatomical structure are described for the most most positive and most negaive ends of a given axis. Statistical outputs for each assoication are listed in the legends.
